# Heat-Induced Discoloration of Chromophore Structures in Eucalyptus Lignin

**DOI:** 10.3390/ma11091686

**Published:** 2018-09-11

**Authors:** Peng Zhang, Yanxia Wei, Yang Liu, Jianmin Gao, Yao Chen, Yongming Fan

**Affiliations:** MOE Key Laboratory of Wooden Material Science and Application, College of Materials Science and Technology, Beijing Forestry University, Beijing 100083, China; rulai9565@hotmail.com (P.Z.); wyx0408@bjfu.edu.cn (Y.W.); nolekobe@sina.com (Y.L.); fanym@bjfu.edu.cn (Y.F.)

**Keywords:** heat-induced, wood discoloration, Eucalyptus, lignin, chromophore system

## Abstract

The color changes corresponding to chromophore structures in lignin caused by exposure of Eucalyptus (*Eucalyptus grandis* and *E. urophylla*) to heat were investigated. Eucalyptus wood powders were heat treated under saturated steam atmospheres for 10 h at 110 °C, 130 °C and 150 °C. The lignin was isolated before and after heat treatment. The physicochemical properties of the lignin and changes in chromophore structures during heat treatment was evaluated through wet chemical analysis, Fourier transform infrared spectroscopy (FTIR), diffuse reflectance ultraviolet-visible spectroscopy (DRUV-Vis), gel permeation chromatography (GPC), X-ray photoelectron spectroscopy (XPS) and ^13^C Cross polarization magic angle spinning nuclear magnetic resonance (^13^C CPMAS NMR). Wood color darkened and reddened with the increase in pressure and temperature. Depolymerization and dehydration reactions occurred via demethoxylation with heat treatment in saturated steam at 110 °C or 130 °C. Lignin condensed to form insoluble compounds after heat treatment in saturated steam at 150 °C. G units increased and S units decreased through demethylation during heat treatment, as revealed by FTIR and ^13^C-NMR analysis.

## 1. Introduction

Wood is a popular decorative material due to the structure and texture, especially its natural color. Wood products with red and dark hues are especially favored by consumers. The heat-induced coloring of wood is of greater interest to consumers, due to the global tendency of curtailment of solvent-based coatings in furniture processing [1]. The addition of no harmful additives and the improvement of color by changing the chromophoric system of wood during heat treatment is an environmentally friendly, stable method [2]. Heat-induced discoloration technology turns light-colored wood to a red dark hue, thus providing a potential value for fast-growing wood species [3] and the possibility of reaching traditional markets where reddened hardwoods are usually dominated [4,5].

The mechanism of heat-induced discoloration of wood is mainly due to chemical changes of cell wall constituents [5,6,7]. Thermal modifications include decomposition of hemicelluloses [8]; increased crystallinity of cellulose [9]; cleavage of β-O-4 linkages and demethoxylation in lignin, leading to more condensed structures and oxidation products such as quinones [10,11]. The release of acetic and formic acid by carbohydrates could catalyze hydrolytic reactions [12,13] such as the oxidation and polymerization of phenolic compounds and lignin. Discoloration during wood heat treatment depends on heating conditions that include heat medium, temperature, moisture content, humidity, and air pressure. The degree of discoloring increases with the temperature, duration time, moisture content, and the presence of oxygen [8]. Thermal treatment under hot air conditions ranging from 180 °C to 220 °C usually causes degradation of hemicellulose and amorphous areas of cellulose [14,15]. Wikberg [10], using steam as a shielding gas to treat wood samples in 160 °C and 195 °C under normal pressure, achieved the same results as thermal treatment [10]. Vegetable oil was also used as a medium in heat treatment to exclude oxygen impact and better dimensional stability and photostability were obtained [16]. Chen et.al. used saturated steam at 130 °C to induce discoloration, with no change in the structure of cellulose and hemicellulose [2,17,18], demonstrating that higher humidity can cause obvious discoloration under saturated steam treated especially at lower temperature, with no effect on polysaccharide components. Although the saturated steam method effectively induces discoloring, it has received less attention by the wood industry and should be considered. Color changes are presumably more linked to changes in lignin substance than in the carbohydrate fractions [3]. Vacuum drying of Brauns’ lignin revealed that color change is affected by the variation of Brauns’ lignin structure [19]. Conjugated structures formed and generally lead to color changing in the process of lignin degradation under heat treatment [20]. Functional groups in lignin, such as α-carbonyl, biphenyl and ring-conjugated double bonds, are known to absorb UV-light and form chromophores [21]. Quinone and conjugated carbonyl are the main chromophoric groups, and phenol hydroxyl is the main auxochrome group in wood [22]. Analysis of heat effects in the discoloration mechanism must involve not only wood, but also its isolated lignin.

We here report our study of the effects of steam heat treatment on lignin (especially chromophore structure and content) and its relationship to the discoloration mechanism, and the role of the corresponding changes of the chromophores in the color of wood.

## 2. Materials and Methods

### 2.1. Wood Samples and Thermal Modification

*Eucalyptus grandis* and *E. urophylla* wood logs were collected from Guangxi Province, southwest China. Wood logs were cut and grounded into powder to be analyzed. The wood powder (150 μm-sieved) was air-dried to a moisture content of 7–12% before heat treatment. The extractives were removed completely using benzene/alcohol (*v*/*v*, 2/1) in a Soxhlet extractor for 24 h. After extraction, the wood samples were dried for 2 h at 103 °C and then put in a desiccator until further analysis. Heat treatments were performed in the extractive-free wood powders in saturated steam atmosphere for 10 h at 110 °C (0.15 MPa), 130 °C (0.27 MPa) and 150 °C (0.49 MPa) in a Steam Autoclave (Binjiang Medical LS-35HD, Jiangyin, China).

### 2.2. Color Measurement

A DF110 spectrophotometer (Konica Minolta CM-2300d, Tokyo, Japan) was used to collect the colorimetric data according to the CIEL*a*b* system [23,24] using 10° standard observer and standard illuminant D65. The spectrophotometer is a small handheld instrument with an aperture size of 8 mm. Wood powder were evenly laid on a flat surface when testing. Five measures were read per sample. The color coordinates lightness L* (varying from 0 for black to 100 for white), a* (varying from negative values for green to positive values for red on the green–red axis) and b* (varying from negative values for blue to positive values for yellow on the blue–yellow axis) were measured. Color measurements were performed on three points for each sample and an average value was calculated. Color differences of the samples before and after heat treatment were calculated based on the following Equations (1)–(4):

(1) $$\Delta L^{*}=L_{\mathrm{heated}}^{*}-L_{\mathrm{unheated}}^{*}$$

(2) $$\Delta a^{*}=a_{\mathrm{heated}}^{*}-a_{\mathrm{unheated}}^{*}$$

(3) $$\Delta b^{*}=b_{\mathrm{heated}}^{*}-b_{\mathrm{unheated}}^{*}$$

(4) $$\Delta E^{*}=\sqrt{\Delta L^{*2}+\Delta a^{*2}+\Delta b^{*2}}$$

where ΔL*, Δa*, Δb* and ΔE* represent the variations in the lightness, green/red coordinate, blue/yellow coordinate, and total color difference, respectively.

DRUV-Vis (diffuse reflectance ultraviolet-visible spectroscopy) were recorded over the wavelength range of 200–700 nm on a UV-vis spectrophotometer (Shimadzu UV-2550, Kyoto, Japan) equipped with an integrated sphere. BaSO_4_ was used as a reference sample. Wood powder were made into thin slices (30 mm diameter) by EVA (ethylene-vinyl acetate copolymer) hot glue so that the loose powder can be detected in the spectrophotometer.

### 2.3. Lignin Analysis

The content of Klason (acid-insoluble) lignin was determined according to ASTM D 1106-96 [25]. Extractive-free wood powder (500 g) was first hydrolyzed with 72% sulfuric acid (7.5 mL) for 2 h at 20 °C. Distilled water was then added to decrease the acid concentration to 3% (*w*/*v*) and the hydrolysate was boiled for 1.5 h in autoclave at 121 °C. The hydrolysate was then washed three times with hot distilled water to pH 7.0 and filtered with glass crucibles (1G3). Finally, the Klason lignin was obtained as residue and dried for further analysis. The lignin structures before and after heat treatment were determined on dioxane lignin [26,27]. The dioxane lignin was prepared using the method reported by Evtuguin et al. [28]. The wood flour was placed in a flask and heated in a water bath at 95 °C for 2 h in a solvent containing a mixture of hydrogen chloride in dioxane/water (9:1, *v*/*v*). This process was repeated three times. The extract was concentrated and added to distilled water to precipitate lignin.

Elemental analysis and content of the dioxane lignin was analyzed on a Thermo Scientific Flash 2000 series elemental analyzer. Oxygen content was calculated by subtracting the content of C, H, N, and S from 100% [29,30]. The molar ratio of O/C and H/C was calculated based on Equation (5,6), separately.

(5) Mol(O)/Mol(C)=W%(O)/M(O)W%(C)/M(C)

(6) Mol(H)/Mol(C)=W%(H)/M(H)W%(C)/M(C)

where M(O), M(H), M(C) represent the molar mass of O, H and C, separately.

FTIR (Fourier transform infrared spectroscopy) spectra were recorded in absorbance mode using a PERKIN Elmer Spectrum Gx instrument (Perkin Elmer, Shanghai, China). The concentration of lignin in a KBr pellet was about 1%. The number of scans was 32, the resolution was 4 cm^−1^, and the sweep scan range was 400–4000 cm^−1^. The relative peak height (H’) of carbonyl groups was calculated based on Equation (7):

(7) H′=HH(1507)

where H (1507) represent the intensity of 1507 cm^−1^ in aromatic skeleton; H and H′ represent the intensity and relative intensity of carbonyl groups.

The dioxane lignin was acetylated with acetic anhydride/pyridine (1:1, *v*/*v*) at 50 °C for 72 h in a sealed container. Ethanol was added, and the volatiles were removed in a vacuum evaporator; the procedure was repeated three times. A gel permeation chromatography (GPC) apparatus with THF (tetrahydrofuran) as eluent at a flow rate of 1.0 mL/min at 25 °C was used to measure the average number of molecular weights (Mn) and molecular weight distributions (PDI), which was calibrated by using linear polystyrene standards [30,31].

^13^C CP-MAS NMR (^13^C Cross Polarization Magic Angle Spinning Nuclear Magnetic Resonance) spectroscopy was recorded using a JNM-ECZ600R NMR spectrometer (JEOL, Tokyo, Japan) with a magnetic flux density of 14.09 T. Carbon spectra were acquired with a 4 mm bore probe head. The spinning rate was set to 15 kHz. One thousand and twenty-four scans with 3 s delay between successive scans were collected. The length of the contact time was 2 ms, and the spectral width was 37.89 kHz.

XPS (X-ray photoelectron spectroscopy) analysis was performed on dioxane lignin with a ThermoVG Scientific Sigma Probe (Thermo Fisher ESCALAB 250Xi, Waltham, MA, USA) using a microfocusing monochromatic AlKa X-ray source at an operating pressure between 10–9 and 10–8 mbar. A high-resolution scan was conducted on the C_1S_ peak from 280 to 300 eV, and the O_1S_ peak from 530 to 535 eV for each sample. Chemical bond analysis of carbon was accomplished by fitting the C_1S_ peak and deconvoluting into four sub peaks for C-C/C=C, C-O, and C=O- groups, with areas represented by C_1_, C_2_, C_3_, respectively. The O_1S_ signals were similarly deconvoluted into two sub peaks for C=O and C-O groups and represented by O_1_ and O_2_, respectively. The ratio of C_3_/C_2_ and O_1_/O_2_ was calculated [32].

## 3. Results

### 3.1. Heat Treatment Effects on Color Parameters of Wood Samples

**Figure 1:**
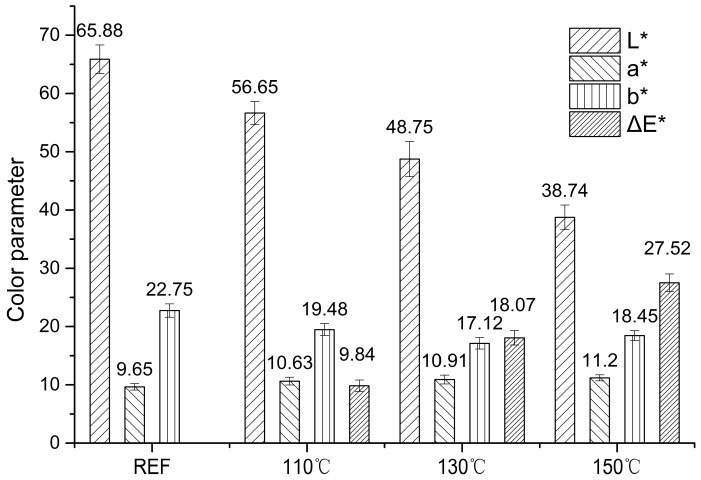
Comparative color parameter analyses under saturated steam.

A summary of color parameters (average values of L*, a*, b* and calculated ΔE*) for the extract-free treated samples are presented in Figure 1. All samples underwent an obvious color change that was perceived visually as darkening and reddening. This demonstrated that saturated steam heat treatment was an effective induced discoloration method. The ΔE* value indicated the degree of color change that was elevated dramatically with increasing temperature and pressure, similar to superheated steam conditions [33,34], which implied that visible light absorption effect enhanced in the wood surface during this process [17]. L*, which was the most sensitive parameter, decreased 20% in each treatment. The parameter a* value, which caused the samples to redden, increased along with the severity of the heat condition and quickly achieved the maximum value, then rose slightly. It is usually revealed that condensation and oxidation products, such as the quinone [13], absorb the complementary light of reddish color in the visible spectrum [2]. The parameter b* value changed in a small range after heating and varied below 20. The gradually higher yellowness treated under 150 °C could be partly caused by formation of oxygen-containing groups such as carbonyl, carboxyl and hydroperoxide groups, which are mostly pale-yellow substances [35,36].

**Figure 2:**
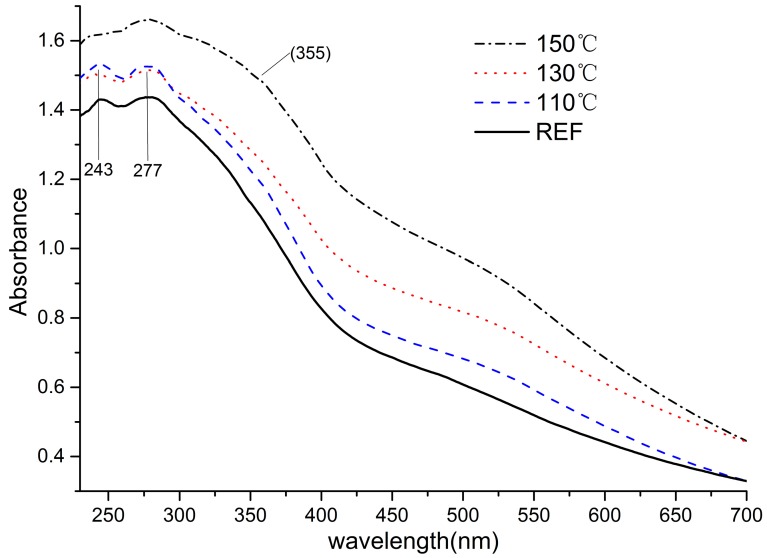
DRUV-Vis spectrum of wood flours treated in saturated steam.

Light absorption coefficient spectra can be used to identify qualitative increases in the chromophoric and leucochromophoric structures [37]. DRUV-Vis spectra obtained from the extractive-free samples heated at various conditions are shown in Figure 2. The absorptions of treated samples increased obviously in the visible spectrum, which indicated that chromophoric reactions occurred and intensified during steam heat treatment. The color variation can be characterized directly by the reflections in the visible region (380–780 nm). The absorbance in the visible region of the sample heat-treated at 110 °C increased compared to the reference curve; the absorbance was stronger when the sample heat treated at 130 °C. The increase in absorbance in the region of 380–550 was vigorous when heat treated at 150 °C, which corresponds to the color parameter changes.

For further explanation of discoloration, reflections in the UV region were evaluated. Two peaks (244 nm and 277 nm) intensified with the severity of heat condition. The peak at 244 nm belongs to π→π* (B band) absorbance that represents the benzene ring vibration of the aromatic compounds. The peak appearing near 277 nm belongs to the n→π* (R band) absorption, which was the chromophore vibration. In the case of lignin, these signals specify the existance of non-conjugated phenolic groups and unsaturated Cα, Cβ bonds and β-C = O structures [38]. Samples treated at 110 °C and 130 °C showed the same absorbance in 244 nm and 277 nm peaks, but differed in the visible region, which implies that discoloration at 110 °C and 130 °C did not originate from chromophoric reactions. Absorbance of these two peaks increased in the 150 °C curves, demonstrating that vigorous chromophoric reactions occurred in this condition, explaining the increase in absorption and color parameters under the 150 °C-saturated steam treatment. The spectrum region in 300–400 nm intensified with increasing temperature; the subtle shoulder at 355 nm resulted from conjugated structures (double bonds and the conjugated carbonyl group in lignin side chains). Quinoid structures, which formed in the process of hydroxyl oxidation in lignin aromatics, could have also contributed to the subtle shoulders among 300–400 nm.

### 3.2. Content and Elemental Composition of Lignins

After the wood powders were treated and the color variation was evaluated, the lignins were extracted for further study. For the first step, the content changes of acid-insoluble lignin (Klason lignin) and element changes of dioxane lignin were analyzed (Table 1). The Klason lignin content variation showed the same direction with the dioxane lignin, which increased for 110 °C and 130 °C conditions and then decreased nearly to the original level in 150 °C treatments.

The increased trend has been observed in many heat treatment studies [39,40,41] and is likely due to demethoxylation. At the same time, the O/C ratio was slightly increased, implying that oxidation reactions occurred in lignin during the heat process. Oxidation reactions possibly coincided with demethylation in the C–3/5 position, therefore leading to depolymerization between lignin structural units.

The reduction trend was rarely reported in previous studies. The decreased lignin content and the corresponding O/C ratio under the 150 °C conditions could be explained by condensation of acid-insoluble lignin, followed by reduction reactions. The decrease of O/C revealed e hydroxyl groups loss when the dehydration reactions occurred and removal of methoxyl groups from lignin condensation [42], which also led to the decrease of hydrogen and oxygen content. Nitrogen in the samples may have originated from a grain of proteins in wood which possibly strongly bound to the lignin [43]. Both lignin contained small amounts of sulfur, possibly due to contamination during the washing step. The 150 °C-treated condition can be viewed as a turning point for the different trends in 110 °C and 150 °C conditions.

### 3.3. GPC Analysis of Molecular Weight and Polydispersity Index

For in-depth studies of structure changes of lignin after treatment, average molecular weight (Mw and Mn) and polydispersity index (PDI) was demonstrated by GPC analysis (Table 2). The Mw of the reference sample was 7025, which was close to the literature reported value of 7590 of MWL (Milled Wood Lignin). The molecular weight of lignin had two stages of variation. Groups in the 110 °C and 130 °C conditions can be seen as the first stage in which PDI increased, while Mw decreased 56% and 64%, respectively, as compared to the reference lignin. A part of lignin was degraded, leading to an uneven distribution of molecular weight. The decrease in hydroxyl groups in lignin, as aforementioned with increasing temperature, was consistent with the decreased molecular weight [44].

The second stage occurred in the 150 °C group where higher Mw (8777) and polydispersity index (2.026) indicated condensation in parts of the lignin, which led to increased molecular weight. Therefore, the molecular weight distribution tends to become even and PDI decreased almost to the original level in the process, which is in accordance with the lignin analysis. In summary, condensation [45] and oxidation were the main reactions in the first stage (110 °C and 130 °C), whereas depolymerization and reduction were predominant in the second stage when the temperature increased to 150 °C.

### 3.4. FTIR Spectra of the Lignin

**Figure 3:**
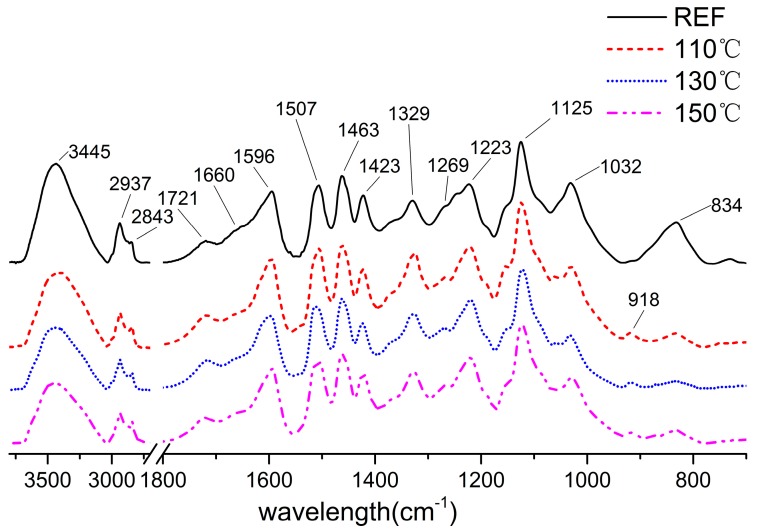
The FTIR spectra of dioxane lignin extracted from samples untreated and treated.

Fourier-transform infrared (FTIR) spectroscopy was used to investigate the changes of functional groups. The FTIR spectra of dioxane lignin was normalized at 1507 cm^−1^ (Figure 3), for the aromatic ring vibration around 1510 cm^−1^ was stable during treatment [46].

The decreased intensity of OH stretch vibration at 3445 cm^−1^ implied the reduction of hydroxyl in lignin, such as the C-4 and C-γ positions that were also accompanied with condensation of lignin. The intensity in 2843 cm^−1^ (CH_2_ stretch vibration) increased compared to the reference lignin could be caused by the extended side chain in lignin. The slightly increased vibration of non-conjugated C=O at 1721 cm^−1^ implied the existence of carboxyl groups and ester structures which would result in the darkening of treated powders. The relative peak height (H′) of carbonyl groups, representing the relative content of carbonyl groups, were listed in Table 3.

The content of non-conjugated C=O (1721 cm^−1^) first increased and then decreased, showing the same trend of variation in lignin content and its molecular weight. Whereas the absorption band at 1660 cm^−1^, associated with conjugated C=O groups, showed a slight decrease after heat treatment, originating from the depolymerization of lignin. The decreasing of non-conjugated C=O groups was probably caused by the condensation reactions of lignin [45]. We can conclude that the oxidation reaction led to the formation of non-conjugated carbonyl and did not affect conjugated carbonyl groups. After heat treatment, the vibration at 1596 cm^−1^ and 1507 cm^−1^ showed stable aromatic structures during the process. The vibration at 1507 cm^−1^ was especially difficult to change in different heat treatments. The slightly intensified peak at 1329 cm^−1^ implied that the condensation degree of lignin increased during the process. The bands that are associated with guaiacyl units (1269 cm^−1^, C-O stretch in OCH_3_; 1125 cm^−1^; 1032 cm^−1^, C-H in-plane deformation; 918 cm^−1^, C-H aromatic out-of-plane deformation) increased after heat treatment, signifying more formation of guaiacyl units which possibly come from the syringyl units through demethoxylation. These results are also consistent with bands from syringyl units (1220 cm^−1^, C-O stretch; 834 cm^−1^, C-H out-of-plane in position 2 and 6) that decreased after treatment. According to Faix [47], the Eucalyptus dioxane lignin can be classified as a GS4 type whose constituent ratio is H (p-hydroxyphenyl units 0–5%), G (guaiacyl units 25–50%), S (syringyl units 45–70%), and OMe/C900 (135–170). Among various types of lignin, the GS4 type contains the lowest content of G units. In the present study, S unit content decreased because of demethoxylation during the heat treatment.

### 3.5. ^13^C NMR Spectroscopy

**Figure 4:**
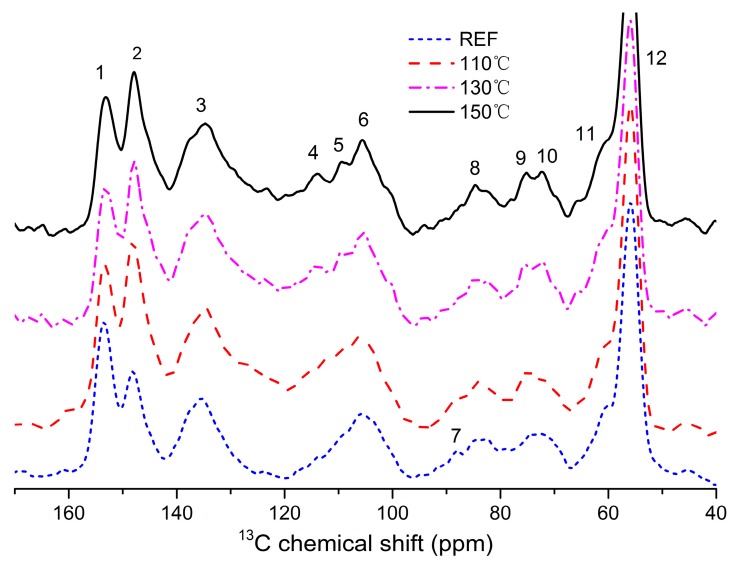
The ^13^C NMR spectrum of dioxane lignin isolated from treated wood samples.

^13^C NMR spectroscopy is a non-destructive technique for obtaining detailed information about lignin structures [30]. The ^13^C NMR spectrum of dioxane lignin is given in Figure 4.

The chemical shifts (ppm), intensity, and assignments as summarized in Table 4, are the results of many previous studies [4,15,48,49]. As shown in Table 4, typical polysaccharide signals between 57 and 103 ppm (which usually exists in 65, 72, 89, 102, 173 ppm) were nearly absent in the lignin, implying that only a trace number of associated polysaccharides left the lignin preparation. The syringyl (S) residues were assigned at 153, 148, 105 and 135 ppm. Guaiacyl (G) remines were validated by signals at 148, 135, and 105 ppm. The p-hydroxyphenyl (H) remines, assigned at 128 ppm (C-2/C-6) [48], were not detected. These signals revealed that the lignin fraction could be verified as GS lignin, which consistent with the results obtained in FTIR analysis. The signal intensities were considered to be semiquantitative. The percentage values (%) of carbons are calculated from integrated intensities of signals within each shift range. Using an integral between 160–96 ppm regions as the reference, it assumed to include six aromatic carbons and 0.12 vinylic carbons. It follows that the integral value (160–102 ppm region) divided by 6.12 is equivalent to one aromatic ring (Ar) [50,51]. The relative signal intensities were compared in Table 4.

The aromatic part of lignin was shown in the region between 102 and 162 ppm, and can be further split into three zones: protonated aromatics (δ 123–102 ppm); condensed aromatics (*δ* 140–123 ppm); and oxygenated aromatics (δ 162–140 ppm) [52]. Therefore, the differences of the zone integral can determine the degree of condensation of lignin. The oxygenated aromatic region shows a higher intensity at 153 ppm (related to syringyl) than at 148 ppm (related to guaiacyl) in the reference lignin, which reveals that S units are more than G units linking with other lignin units. The peak at 153 ppm intensity reduced and was lower than that of 148 ppm after heat treatment, showing the cleavage degree of the β-O-4 linkages, which results in a decrease in etherified syringyl-propane units [48] and an increase in phenolic structure during the process. A considerable number of the β-O-4 linkages were cleaved, mainly due to the saturated steam treatment [53,54]. This decrease observed for the intensity at 153 ppm may have also resulted from the depolymerization of lignins when β-O-4 bonds cleaved (which is not the predominant chemical route) and the demethoxylation process which S units (syringyls) become guaiacyl units. Demethoxylation seems to be an important process for explaining most of this variation since signal 12 (56 ppm represented methoxyl groups), decreased with increasing temperature, which preferentially reflects one site (C1/C4) of the syringyl units [15]. Demethoxylation of lignin leads to a condensed lignin structure (obtained in the GPC results) and more reactive sites in lignin.

Heat treatment in saturated steam depolymerized wood lignin partially by hydrolyzing the aryl ether linkages involving C-4 syringyl and guaiacyl units, in which free phenolic hydroxyl groups formed and more α- and β-carbonyl groups may have possibly appeared. However, carbonyl groups (196–193 ppm) signal did not appeared in the spectra. It is possible that some of the volatile and water-soluble products, such as depolymerized lignin, are leached from the samples with steam, and the remaining amount is too low to be detected by solid state nuclear magnetic resonance [10].

The peak at 135 ppm was classified as condensed aromatics region which, assigned to C1/C4 sites of etherified S and G units, was steady during steam treated process. This region was comprised C-1 carbons and other aromatic carbons involved in crosslinking, such as the 5-5 or β-5 substructures. The peak at 105 ppm can be classified as protonated aromatics region which rarely change. The appearance of β-O-aryl ether structure in dioxane lignin (Figure 4) was identified with three peaks at 85, 75 and 60 ppm (corresponding to aliphatic carbons bound to oxygen) [55,56,57], which assigned to C-β, C-α, and C-γ in β-O-4 structures, respectively. This intensity of these three peak β-O-aryl ether structures reveals that the treated process did not significantly cleaved the β-aryl ether structure. Also, it is shown that dioxane lignin is a suitable choice for analysis of the present lignin structure.

### 3.6. XPS Analysis

**Figure 5:**
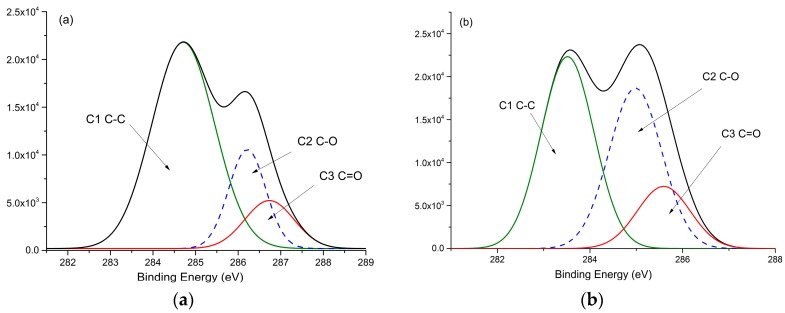

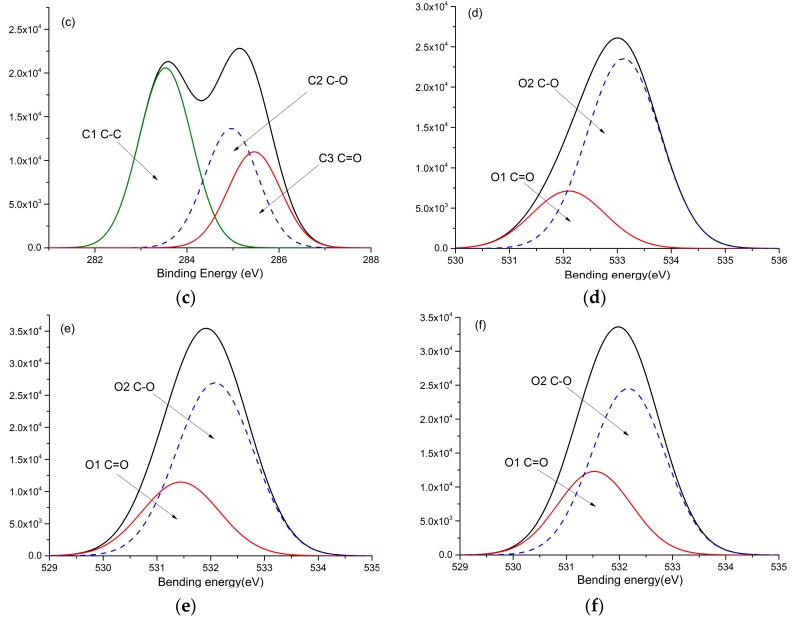
X-ray photoelectron spectroscopy scan of C_1S_ and O_1S_ of lignin isolated from treated wood samples: (**a**) Curve fitting of the C_1s_ peak of reference samples; (**b**) Curve fitting of the C_1s_ peak under 130 °C; (**c**) Curve fitting of the C_1s_ peak under 150 °C; (**d**) Curve fitting of the O_1s_ peak of reference samples; (**e**) Curve fitting of the O_1s_ peak under 130 °C; (**f**) Curve fitting of the O_1s_ peak under 150 °C.

Figure 5 shows the high-resolution spectra of C_1S_ carbon and O_1S_ oxygen. Table 5 presents the binding energies of C_1_, C_2_, C_3_, O_1_, O_2_, plus the C_3_/C_2_ and O_1_/O_2_ ratios. The relative area of C_4_, representing carboxylic functionalities, were not detected in the lignin XPS spectrum, which suggests a very low content of carboxylic groups in lignin.

The carbonyl structure produced after heat treatment, as demonstrated by an increase in the ratios C_3_/C_2_ and O_1_/O_2_, is largely due to an increase in C_3_ and O_1_ representing non-conjugated C=O groups and is confirmed by the intensified peak at 1721 cm^−1^ in FTIR spectra. A similar trend to the lignin content and ratio of O/C appears in C_3_, that increased for the 110 °C and 130 °C conditions but decreased in 150 °C conditions, imply that various C-O groups affect the PDI, content and O/C ratio of lignin. It can be confirmed that the carbonyl structure contributes to the chroma value of a* which increased during the heat treatment. The presence of carbonyl structures and quinones are the reason for the a* value [2], which is consistent with the increased chroma value of a* for different heated samples. The chroma value of b* is influenced by the C-O groups and shows the opposite trend to O/C ratio of lignin. This can be a more obvious indication for chromophore variation.

## 4. Conclusions

The mechanism of steam heat-induced discoloration was investigated via analyzing chromophoric structures in lignin structures. Wood color becomes darker and redder with increasing pressure and temperature; chromophoric reactions in lignin intensify with the severity of conditions. Non-conjugated carbonyl groups and unsaturated Cα, Cβ bonds, and β-C=O structures increase in the heating process. Under 110 °C and 130 °C steam heat treatment, depolymerization in lignin forms via demethoxylation. In 150 °C steam treatment, there is a conversion of condensed and dehydration reactions, and the acid-soluble lignin condenses to form insoluble compounds. G units increase and S units decrease through demethylation during the treatment, as confirmed by FTIR and C NMR analysis. Carbonyl structure contribute to the chroma value of a* which increases during the heat treatment. The chroma value of b* is influenced by the C-O groups and show the opposite trend to O/C ratio of lignin.

## Figures and Tables

**Table 1:** Chemical composition of lignin during the heat treatment.

T (°C)	Klason Lignin (%)	Dioxane Lignin (%)							
	Content	Content	C	H	O	N	S	O/C	H/C
REF	22.66 (±0.34)	6.06 (±0.41)	57.25	5.75	36.79	0.08	0.131	0.48	1.20
110	24.17 (±0.21)	11.24 (±1.1)	56.06	5.35	38.29	0.10	0.193	0.51	1.15
130	25.4 (±0.52)	12.2 (±1.8)	55.43	5.19	38.33	0.17	0.884	0.52	1.12
150	21.18 (±0.65)	8.08 (±0.13)	59.02	5.77	34.90	0.12	0.195	0.44	1.17

**Table 2:** Average molecular weight and molecular weight distribution.

Group	Mw (g/mol)	Mn (g/mol)	PDI (Mw/Mn)
REF	7025	3822	1.838
110	3962	1483	2.671
130	4513	1687	2.675
150	8777	4332	2.026

**Table 3:** Relative peak height (H’) of carbonyl groups in the lignin FTIR spectra.

Wave Number	REF	110	130	150
1660(cm^−1^)	0.44	0.34	0.39	0.37
1721(cm^−1^)	0.29	0.32	0.35	0.31

**Table 4:** Assignments of the Lignin ^13^C Signals in the Spectra of the dioxane lignin.

	ppm	Assignment	Amount (Per Ar)			
			REF	110	130	150
1	153.18	C-3, S etherified	1.71	1.05	1.02	0.91
2	148.05	C-3/5, S non-etherified; C-4, G etherified	1.21	1.27	1.27	1.28
3	134.88	C-1/4, S/G etherified	1.47	1.32	1.35	1.44
4	114.2	C-3/5, H etherified	0.23	0.40	0.39	0.40
5	109.61	C-2, G	0.43	0.42	0.42	0.42
6	105.7	C-5/6, G etherified; C-2/6, S etherified	0.79	0.58	0.57	0.66
7	88.16	C-α, phenylcoumarans	0.21	0.13	0.13	0.12
8	85.02	C-β, β-O-4	0.56	0.33	0.35	0.36
9	75.15	C-a, β-O-4	0.31	0.22	0.24	0.27
10	72.28	C-γ, pinoresinols	0.39	0.22	0.21	0.25
11	60.83	C-γ in β-O-4	0.67	0.45	0.43	0.58
12	56	methoxyl groups OCH3	2.53	1.49	1.41	1.65

**Table 5:** Subpeak area fractions of C_1S_ and O_1S_.

Sample Description	Relative Area of C_1s_ Peaks (%)				Relative Area of O_1s_ Peaks (%)		
	C_1_ (C-C/C=C) (284.7 eV)	C_2_(C-O) (285.9 eV)	C_3_(C=O) (286.4 eV)	C_3_/C_2_	O_1_(C=O) (531.7 eV)	O_2_(C-O) (532.8 eV)	O_1_/O_2_
REF	57.32	32.84	9.83	0.30	23.20	76.79	0.30
130	46.83	38.20	14.97	0.39	29.83	70.17	0.43
150	45.39	37.40	17.21	0.46	31.80	68.20	0.47
